# Toxicity of Nanoemulsified *Eugenia uniflora* (Myrtaceae) Essential Oil to *Spodoptera frugiperda* (Lepidoptera: Noctuidae) and Selectivity to *Trichogramma pretiosum* (Hymenoptera: Trichogrammatidae)

**DOI:** 10.3390/plants15020248

**Published:** 2026-01-13

**Authors:** Júlia A. C. Oliveira, Karolina G. Figueiredo, Letícia A. Fernandes, Vinícius C. Carvalho, Dejane S. Alves, Julio C. Ugucioni, Jhones L. Oliveira, Hudson W. P. Carvalho, Suzan K. V. Bertolucci, Geraldo A. Carvalho

**Affiliations:** 1 Departament of Entomology, Federal University of Lavras, Lavras 37203-202, MG, Brazil; julia.assuncaooliveira@hotmail.com (J.A.C.O.); kgfigueiredo17@gmail.com (K.G.F.); leticiafrbs@gmail.com (L.A.F.); vicarvalho06@gmail.com (V.C.C.); 2 Agronomy Course Coordination, Federal Technological University of Paraná, Santa Helena 85892-000, PR, Brazil; dejanealves@utfpr.edu.br; 3 Department of Physics, Federal University of Lavras, Lavras 37203-202, MG, Brazil; julio.ugucioni@ufla.br; 4 Environmental Nanotechnology Laboratory, Institute of Science and Technology, Paulista State University (ICT/UNESP), Sorocaba 18087-180, SP, Brazil; jholuisoliveira@hotmail.com; 5 Center for Nuclear Energy in Agriculture, University of São Paulo (CENA/USP), São Paulo 05508-220, SP, Brazil; hudson@cena.usp.br; 6 Department of Agriculture, Federal University of Lavras, Lavras 37203-202, MG, Brazil; suzan@ufla.br

**Keywords:** fall armyworm, nanotechnology, bioinsecticides, parasitoid, selectivity, ecotoxicology

## Abstract

*Spodoptera frugiperda* (fall armyworm) is a polyphagous pest with widespread resistance to synthetic insecticides, while essential oils (EOs) and biological control agents, such as the parasitoid *Trichogramma pretiosum*, represent promising strategies in integrated pest management (IPM) programs. This study evaluated the toxicity of *Eugenia uniflora* EO, popularly known as pitanga EO, and nanoemulsion (NEO) to *S. frugiperda* and the selectivity of the NEO to *T. pretiosum*. The EO of *E. uniflora* was characterized by GC-MS/DIC and then diluted in water and Tween 80^®^ for bioassays to estimate the LC_50_ against *S. frugiperda* in Potter’s tower. The NEOs were produced by high-shear dispersion using an Ultra-Turrax and characterized for thermal stability, particle size, polydispersity index (PDI), zeta potential (ζ), temporal stability, and morphology. The NEO was diluted to the LC_50_ (36.05 mg/mL) in 1% Tween 80^®^ solution and tested for toxicity to *S. frugiperda* and to the parasitoid. The majority compounds in the EO from *E. uniflora* were curzerene (34.07%), selina-1,3,7(11)-trien-8-one (10.51%), germacrene B (9.51%) and germacrene D (5.03%). The NEO stored at 25 °C remains stable for up to 30 days after preparation. In addition, the NEO showed a particle size of 283.2 nm, a PDI of 0.289, and a zeta potential (ζ) of −23.2 mV. The *E. uniflora* EO and NEO at a concentration of 36.05 mg/mL were toxic to *S. frugiperda* (36% probability of survival). Furthermore, NEO was selective for *T. pretiosum* in its immature stages. The NEO proved to be stable, effective, and selective, indicating potential for IPM. However, validation under semi-field and field conditions is still necessary.

## 1. Introduction

*Spodoptera frugiperda* (Smith, 1797) (Lepidoptera: Noctuidae) is one of the most destructive agricultural pests worldwide, due to its highly polyphagous behavior, wide host range, remarkable dispersal capacity [1,2,3,4,5], and great adaptability have made *S. frugiperda* one of the most damaging pests in Brazilian agriculture, affecting major crops such as maize, soybean, cotton, and rice [6,7].

Current management strategies for *S. frugiperda* rely mainly on synthetic insecticides and genetically modified *Bt* crops. However, the intensive and often indiscriminate use of these tools has resulted in the selection of resistant populations, environmental contamination, and risks to human health and non-target organisms [8,9]. These limitations have stimulated increasing interest in sustainable and ecologically sound pest management approaches within an integrated pest management (IPM) framework [10,11].

Among alternative strategies, biological control using egg parasitoids of the genus *Trichogramma* has proven to be highly effective. *Trichogramma pretiosum* Riley, 1879 (Hymenoptera: Trichogrammatidae) is native to Brazil, widely distributed throughout the Neotropical region, and commercially produced for use in biological control programs [12,13,14]. This species plays a key ecological role by suppressing pest populations at the egg stage and is one of the most extensively used parasitoids in IPM systems. Therefore, evaluating the selectivity of pest control products toward *T. pretiosum* is essential to ensure the compatibility of chemical or botanical inputs with biological control agents [15,16].

In this context, essential oils (EOs) have emerged as promising alternatives for insect pest management due to their complex chemical composition, multiple modes of action, biodegradability, and generally low toxicity to non-target organisms, including parasitoids such as *T. pretiosum* [17,18,19,20]. However, their practical application is often limited by physicochemical constraints such as low water solubility, high volatility, and chemical instability [21].

In recent years, nanoemulsion technology has gained increasing attention as an effective strategy to overcome these limitations. Nanoemulsions enhance the solubility, stability, bioavailability, and persistence of EOs, while potentially improving their insecticidal efficacy and reducing the required application doses [22,23,24]. Consequently, EO nanoemulsions have been increasingly investigated for agricultural and pest control applications as eco-friendly formulations compatible with IPM principles [25,26].

The EO of *Eugenia uniflora* L. (Myrtaceae), a native Brazilian species, has demonstrated insecticidal, repellent, and toxic effects against several insect pests [27,28,29,30]. Our research group demonstrated the high toxicity of *E. uniflora* OE against *S. frugiperda* caterpillars, highlighting its potential as a botanical insecticide [10]. Additionally, a nanoemulsion formulation of this OE has been developed to enhance its stability and biological performance [31].

Therefore, this study aimed to evaluate the toxicity of *E. uniflora* EO and its nanoemulsion (NEO) against *S. frugiperda*, as well as to assess their physiological selectivity toward the egg parasitoid *T. pretiosum*. By simultaneously addressing insecticidal efficacy and non-target safety, this work contributes to the development of compatible control strategies within IPM programs.

## 2. Results

### 2.1. Chemical Characterization of the EO of E. uniflora

The EO of *E. uniflora* consisted of 31 chemical compounds, three of which were unidentified. The volatile constituents present are hydrocarbon monoterpenes and hydrocarbon and oxygenated sesquiterpenes, which account for 77.03% of the total composition. Oxygenated sesquiterpenes (54.43%) and hydrocarbon sesquiterpenes (28.86%) were the dominant classes present in this EO. The majority of the chemical components were characterized by the presence of the oxygenated sesquiterpenes curzerene (34.07%), Selina-1,3,7(11)-trien-8-one (10.51%), the hydrocarbon sesquiterpenes germacrene D (5.03%), and germacrene B (9.51%), which accounted for 48.61% of the total chemical composition. The other constituents found were (E)-β-ocimene, β- and γ-elemene and (E)-caryophyllene, which varied from 2.53 to 4.95% of the total composition. The presence of rarer constituents, such as viridiflorol, atractylone, germacrone, δ-Cadinene and spathulenol, in the range of 0.73 to 2.90%, was also recorded (Table 1).

### 2.2. Obtaining and Characterizing the NEO of E. uniflora

Based on the estimated LC_50_ (36.05 mg/mL; Table 2) of the EO for *S. frugiperda* caterpillars, the NEO was formulated, and its morphological characteristics were analyzed via scanning electron microscope (SEM) (STEM-FEG) model CLARA.

**Figure 1:**
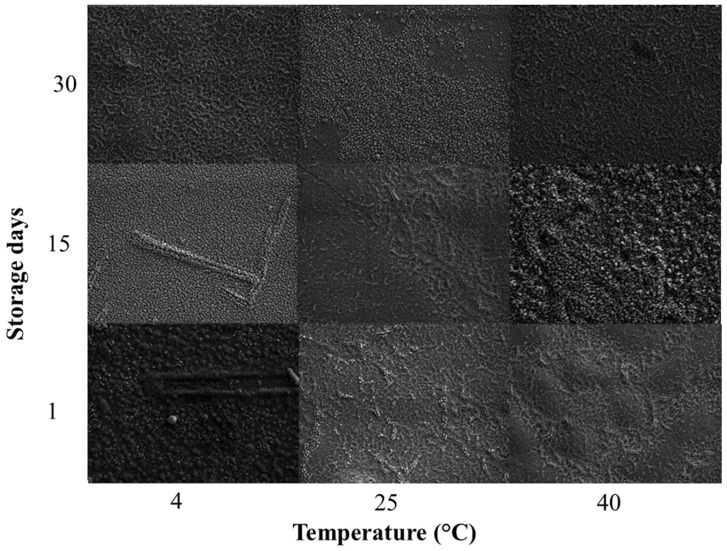
Electron micrographs of nanoemulsions containing essential oil of *Eugenia uniflora* stored at different temperatures and storage periods.

The NEO presented spherical particles on the nanometric scale (Figure 1).

However, these nanospheres of the NEO exhibited variations in mean diameter according to temperature and storage period. The mean diameter of the NEO was evaluated over 1, 15, and 30 days of storage at three temperatures 4 °C, 25 °C, and 40 °C. Significant variations in mean diameter of the NEO were observed as a function of storage temperature, storage time, and the interaction between both factors (df = 4, F = 43.49, *p* < 0.001). After 1 day of storage, the NEO stored at 4 °C had an average size of 27.36 ± 1.04 nm, significantly different from the sample kept at 40 °C (23.86 ± 0.76 nm). After 15 days, the sample stored at 25 °C (22.70 ± 0.75 nm) showed significant differences compared to the other temperatures, while after 30 days all temperatures differed from each other, with average sizes of 19.10 ± 0.47 nm (4 °C), 31.13 ± 0.90 nm (25 °C), and 18.00 ± 0.60 nm (40 °C). These results indicate that both storage time and temperature influence the stability of the mean diameter of NEO (Table 3).

**Figure 2:**
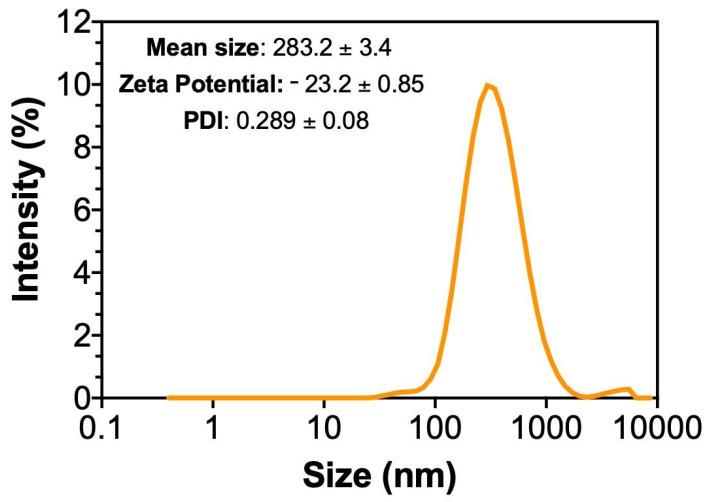
Intensity-weighted particle hydrodynamic diameter distribution (PDI, nm), mean size and zeta potential of NEO of essential oil of *Eugenia uniflora*, ten days after the obtained there.

Dynamic light scattering (DSL) analysis indicated a mean hydrodynamic particle size of 283.2 ± 3.4 nm reflecting the average size of the NEO droplets in suspension. The PDI was 0.289 ± 0.08, and the zeta potential (ζ) was −23.2 ± 0.85 (Figure 2).

### 2.3. Toxicity and Selectivity Bioassays of NEO

#### 2.3.1. Toxicity of EO and NEO to *S. frugiperda*

**Figure 3:**
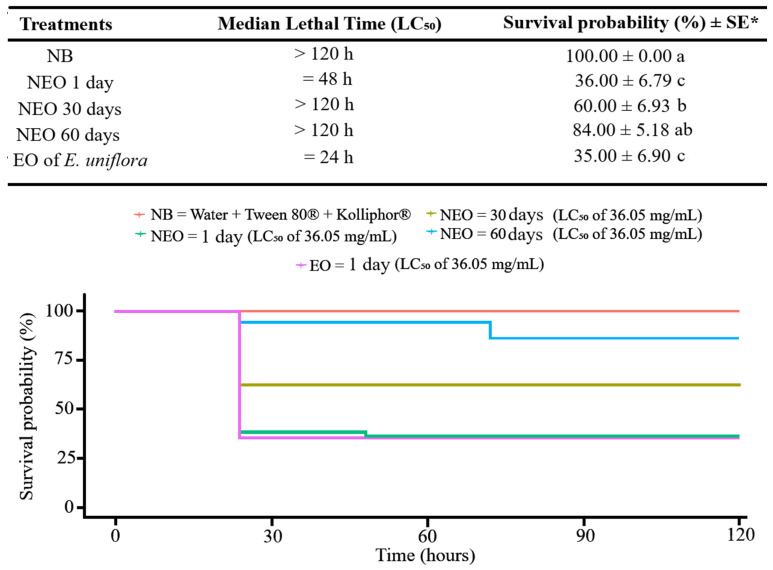
Survival curves estimated using the Kaplan–Meier method of *Spodoptera frugiperda* caterpillars over time following exposure via Potter spray tower to the nanoemulsion diluted to the LC_50_ = 36.05 mg/mL stored for different periods at 25 °C (1, 30, and 60 days). Treatments included: control = nanoemulsion white (NW), and *Eugenia uniflora* essential oil at LC_50_ = 36.05 mg/mL. * Confidence intervals shown; different letters indicate significant differences by log-rank test (*p* < 0.0001).

Given that the NEO stored at 25 °C demonstrated higher stability over 1, 15 and 30, days of storage, its toxicity to *S. frugiperda* caterpillars was subsequently evaluated and compared with that of the EO of *E. uniflora*. The survival probability of caterpillars treated with the NEO (LC_50_ = 36.05 mg/mL) increased with storage time, being 36%, 60% and 84% after 1, 30 and 60 days, respectively. However, the EO of *E. uniflora* at the same concentration (36.05 mg/mL) did not differentiate statistically from the NEO stored for 1 day, with a survival probability of 35% (Figure 3).

#### 2.3.2. Toxicity of NEO to *T. pretiosum*

The NEO did not reduce the emergence of F_1_ parasitoids when exposure occurred during the immature stage (χ^2^ = 333.31, df = 98, *p* = 0.6916), indicating a low impact on parasitoid development under these conditions. The sex ratio of F_1_ adults was also unaffected by NEO exposure (χ^2^ = 333.31, df = 98, *p* = 0.6916). However, in the NEO treatment, parasitism differed significantly between the developmental stages at which the parasitoid was exposed inside the host egg (χ^2^ = 1032.8, df = 76, *p* = 0.0165). F_1_ females originating from host egg treated during the egg–larva period exhibited a significantly higher parasitism rate (21.70 ± 3.27) than those originating from hosts treated during the pupal stage (16.20 ± 3.26), as indicated by different uppercase letters in Table 4. These results indicate that, although NEO exposure affected parasitism, its impact was stage-dependent rather than uniformly detrimental across immature stages. In the F_2_ generation, NEO exposure increased parasitoid emergence by 20% for offspring from the egg–larva (F_1_) treatment (χ^2^ = 365.79, df = 47, *p* = 0.084) and by 12.9% for offspring from the pupa (F_1_) treatment (χ^2^ = 458, df = 43, *p* = 0.082), indicating a positive transgenerational effect (Table 4).

The NEO reduced the parasitism capacity of *T. pretiosum* by 83.80; 91.40 and 81.40% when *E. kuehniella* eggs were treated with NEO and offered after 12, 24 and 48 h, respectively (χ^2^ = 2000, df = 234, *p* > 0.01). A higher percentage of egg parasitism was observed in females from the control after 12 h after treatment (19.50%). Despite the reduction in parasitism, NEO exposure did not affect the longevity of females up to 7 days after contact with treated host eggs. The NEO treatment reduced the emergence of parasitoids in the F_1_ generation (χ^2^ = 647.69, df = 141, *p* = 0.447), with means values ranging from 60.10 to 68.10%. The highest emergence rates in the F_1_ generation were observed in the control treatment (85.00% and 86.40% for eggs treated and offered after 12 and 24 h, respectively). No significant difference was found in the sex ratio of F_1_ individuals that developed in host eggs after 12 and 24 h of exposure (χ^2^ = 788.16, df = 127, *p* > 0.01). However, after 48 h of exposure, the sex ratio in the NEO treatment (0.50) differed from that observed in the control group (0.94). A reduction in parasitism was also observed in females of the F_1_ generation, derived from parasitism by F_0_ females that had contact with NEO 12, 24 and 48 h after treatment of the host eggs, with means of 12.40, 29.03 and 42.97%, respectively (χ^2^ = 1268.1, df = 104, *p* > 0.01). Although NEO exposure caused lower parasitism in F_1_ females, it did not reduce the emergence of F_2_ parasitoids derived from F_1_ females that developed in the host eggs and were offered 48 h later (χ^2^ = 631.53, df = 82, *p* > 0.01). The highest emergence rate was recorded after 12 h of NEO treatment (84.20%), followed by 48 h (48.10%). No significant differences were found in the sex ratio of F_2_ individuals (χ^2^ = 234.21, df = 63, *p* = 0.05), with a predominance of females in all treatments (Table 5).

## 3. Discussion

This study provided novel insights into the toxicity of *E. uniflora* EO and its NEO against *S. frugiperda*, as well as the physiological selectivity of NEO for the parasitoid *T. pretiosum*. Given the scarcity of studies assessing these treatments for both pest and natural enemy, this work represents a pioneering contribution. The results showed that both EO and NEO at a concentration of 36.05 mg/mL were toxic to *S. frugiperda*, while NEO exhibited a low impact on *T. pretiosum* when exposure occurred during immature stages (egg–larva and pupa), as evidenced by minor or no reductions in emergence and the absence of negative transgenerational effects. In contrast, residual exposure to treated host eggs significantly reduced parasitism rates of adult females, indicating the occurrence of sublethal effects depending on the exposure pathway.

Despite the promising insecticidal activity, the EO presents physicochemical limitations such as low vapor pressure and poor aqueous solubility, which can compromise its practical use in crops protection [21,22]. Nanoemulsion technology offers a strategy to overcome these challenges by allowing controlled release of active compounds, improving thermal stability, and prolonging shelf life, while potentially reducing the required dosage [23,24].

The NEO concentration was defined within the range between the LC_50_ and LC_90_ of the EO; however, bioassays were conducted using the LC_50_ (36 mg/mL) estimated in the present study, in accordance with regulatory requirements that target a minimum of 80% pest mortality for product registration [32]. The selection of the LC_50_ concentration for most bioassays was based on its ecological relevance, as it represents a sublethal exposure level commonly used to assess selectivity and sublethal effects on non-target organisms under controlled conditions. This approach allows the detection of physiological and behavioral alterations that may not be evident at higher, fully lethal concentrations. However, we acknowledge that the absence of a full dose–response evaluation for the nanoemulsion represents a limitation of this study. Future investigations should incorporate multiple NEO concentrations, including LC_90_-based treatments, to better characterize dose-dependent effects on both target and non-target organisms and to support more robust risk assessment under integrated pest management scenarios. Microscopic analysis confirmed the formation of spherical nanostructures, consistent with previous studies on NEO of other botanical insecticides [22,33]. The NEO remained stable for up to 30 days at 25 °C, with an average particle size of 283 nm, PDI of 0.289, and ζ potential of −23.2 mV, which indicates adequate colloidal stability under controlled conditions. Differences between SEM and DLS measurements are expected, as SEM provides the physical diameter of individual nanospheres, whereas DLS measures the hydrodynamic diameter of particle dispersed in suspension, including the solvation layer. In contrast, exposure to extreme storage temperatures negatively affected nanoemulsion stability, corroborating precious reports that thermal stress increases system free energy and compromise nanoemulsion integrity [25,26].

The EO composition was dominated by curzerene, with minor compounds including germacrene B and D, (E)-β-ocimene, β-elemene, and E-caryophyllene (1.13–9.51%). The chromatographic profile of the EO of *E. uniflora* observed in this study was consistent with previous reports [10,34,35,36]. Minor variations in EO composition may occur due to factors such as plant age, harvest time, extraction method, cultivation site, and genetic background [21]. Both major and minor constituents are known to contribute to insecticidal activity, likely through multitarget mechanisms affecting metabolic and neural pathways in pests [10,28].

NEO toxicity to *S. frugiperda* was observed, although slightly lower than that of the pure EO at the same concentration. This is consistent with evidence that nanoformulation can modify the bioavailability and biokinetics of active compounds [37,38]. The selectivity for *T. pretiosum* can be attributed to the parasitoid’s limited exposure and developmental stage-specific protection, as eggs contain two chorions during early stages, providing partial shielding from EO contact [39]. However, reductions in parasitism and emergence were observed under certain conditions, such as exposure 48 h post-treatment, suggesting a partial impact likely related to repellent properties of the EO [30,31,39].

Transgenerational effects were minimal when NEO was applied to host eggs containing *T. pretiosum* in the immature stages (egg-larva and pupa), as emergence, parasitism, and sex ratio in the F_1_ and F_2_ generations were not biologically compromised under these conditions. These findings are consistent with previous reports indicating that *E. uniflora* EO and other botanical insecticides may selectively effect on egg parasitoids, particularly when exposure occurs during protected immature stages [40]. However, this outcome does not mitigate the pronounced short-term reduction in parasitism observed in the residual exposure assays, in which F_0_ females exhibited a marked decrease in parasitism capacity within 12–48 h after contact with treated eggs. Although a female-biased sex ratio was observed in some treatments, this outcome alone is insufficient to offset the marked reduction in parasitism caused by residual exposure, reinforcing that population-level impacts must be interpreted by integrating both lethal and sublethal effects. These findings indicate that NEO exerts distinct effects depending on the exposure route and developmental stage, highlighting the importance of differentiating between direct immature-stage exposure and residual adult exposure when assessing selectivity. Therefore, compatibility with *T. pretiosum* should be interpreted as conditional and dependent on application timing, rather than as a full absence of adverse effects.

While the laboratory efficacy of NEO was evident shortly after formulation, its insecticidal activity declines with increasing storage time, indicating limited shelf-life under the tested conditions. Therefore, formulation stability represents a critical factor for practical use. In addition to storage-related degradation, environmental factors such as UV radiation, rainfall, and temperature fluctuations may further reduce effectiveness under field conditions [21,22]. Importantly, the high efficacy observed during the initial storage period highlights the potential of NEO as a viable formulation for practical use when applied within an appropriate shelf-life window. Therefore, further optimization of formulation stability represents a technological challenge rather than a limitation of the bioactive potential of the NEO itself. Additionally, compatibility with standard spraying equipment, formulation costs, regulatory requirements, and persistence in agroecosystems must be evaluated.

In the IPM programs, insecticides should combine effectiveness against target pests with minimal adverse effects on beneficial organisms. The NEO formulation evaluated in this study effectively reduced *S. frugiperda* populations while exhibiting low impact on *T. pretiosum* during its egg-larva period and pupa stage, supporting its potential integration in to IPM programs. However, residual effects on the parasitism capacity of adult females shortly after application highlighting the importance of application timing to minimize disruption of biological control.

From a practical perspective, the results indicate that field applications of NEO would be more compatible with *T. pretiosum* releases if performed between 48 and 72 h after parasitoid release, when residual effects on parasitism are absent or markedly reduced, and egg-larva period are not negatively affected. Alternatively, applications conducted approximately 168–192 h after release, corresponding to the pupal stage of the parasitoid, may also minimize adverse effects. These temporal windows may allow the combined use of NEO and *T. pretiosum* within IPM programs, preserving parasitoid performance while maintaining pest suppression.

Future studies should validate these findings under semi-field and field conditions, evaluate the safety of NEO for other non-target organisms, and assess formulation persistence, cost-effectiveness, and scalability for industrial production. Addressing these aspects will be essential to translate the promising laboratory results into practical and sustainable agricultural applications.

## 4. Materials and Methods

### 4.1. Collection and Chemical Analysis of the EO of E. uniflora

The EO of *E. uniflora* leaves was purchased from TUUA Indústria e Comércio de Cosméticos e Produtos Naturais, Palmas-PR, Brazil. Chemical characterization was performed using gas chromatography techniques, as described by [10]. In the analysis, peaks with a percentage normalized area greater than 1.0% ± standard deviation (n = 3) were considered.

### 4.2. Breeding and Multiplication of S. frugiperda and T. pretiosum

The rearing of *S. frugiperda* was performed as described by [10]. The *T. pretiosum* parasitoids were reared and propagated based on the eggs of the alternative host *Ephestia kuehniella* (Zeller, 1879) (Lepidoptera: Pyralidae), which were fed pure honey and stored in an air-conditioned room at 25 ± 2 °C with a relative humidity of 60 ± 10% and a photophase of 14 h according to [41].

### 4.3. Bioassays with S. frugiperda

#### 4.3.1. Screening of Toxicity of *E. uniflora* EO for *S. frugiperda*

Aliquots of 10, 12 and 15 mg of *E. uniflora* EO were diluted in 10 mL of water and 1% Tween 80^®^ to perform a preliminary screening of toxicity for *S. frugiperda* caterpillars. Then, 2 mL of these solutions was sprayed through a Potter tower (Burkard, Uxbridge, UK) calibrated at 15 lb/in^2^ pressure to deposit 1.5 ± 0.5 μL/cm^2^, as recommended by the IOBC [42], in 72 h-old (2nd instar) *S. frugiperda* caterpillars obtained in laboratory rearing. Soon after spraying, the caterpillars were placed individually in glass tubes (8.5 cm height × 2.0 cm diameter) that contained a piece of artificial diet prepared according to [43]. The bioassay was conducted in accordance with a completely randomized design, with 50 replicates per treatment, each consisting of a single caterpillar. The negative control consisted of an aqueous solution of 1% Tween 80^®^. The number of live insects was evaluated every 24 h for 168 h after treatment. The bioassay was repeated twice on different days.

#### 4.3.2. Estimation of the Lethal Concentration (LC_25, 50 and 90_) of *E. uniflora* EO for *S. frugiperda* via Spraying in Potter’s Tower

Since the *E. uniflora* EO showed acute toxicity to *S. frugiperda* caterpillars in the previous bioassay, five concentrations (30, 50, 80, 125 and 200 mg/mL) were selected to generate ranges that caused mortality from 20 to 80% of the insects [44], to estimate the lethal concentrations (LCs) 25, 50 and 90. The bioassays and monitoring of caterpillar mortality were conducted as described in subitem 4.3.1.

### 4.4. Obtaining and Characterization the Nanoemulsion (NEO) of EO of E. uniflora

The *E. uniflora* NEO was formulated based on the EO LC_50_ and LC_90_ estimated for *S. frugiperda* caterpillar (subitem 4.3.2). The NEO was developed by the authors and is currently under patent protection [31] (protocol No. NBR 1020240072944). The NEO was prepared using a combination of two surfactants in two phases. Phase 1 consisted of Tween 80^®^ (5% *m*/*v*) + ultrapure water (50% *v*/*v*), and phase 2 was composed of *E. uniflora* EO (25% *m*/*v*) + Kholliphor^®^ (5% *m*/*v*) + ultrapure water (20% *v*/*v*). Initially, the NEO phase 1 components were subjected to agitation at 5000 rpm in an UltraTurrax^®^ T18 (São Paulo, Brazil) for 3 min. Next, the phase 2 constituents were gradually incorporated with the phase 1 constituents via an automatic pipette. After this step, the mixture was stirred in UltraTurrax^®^ at progressive speeds of 5000, 10,000 and 15,000 rpm, remaining for 3 min in each speed cycle. Upon reaching maximum rotation speed, the speed was decreased by 5000 at 3 min intervals until the static condition was reached. A blank formulation (control) was also prepared under the same conditions but with only water and surfactants. Thus, in the nanoemulsion white (NW), phase 1 consisted of Tween 80^®^ (6.25% *m*/*v*) + ultrapure water (50% *v*/*v*), and phase 2 consisted of Kholliphor^®^ (6.25% *m*/*v*) + ultrapure water (20% *v*/*v*) [31].

The thermal stability of NEO over time was evaluated at low (4 °C), ambient (25 °C) and high (40 °C) temperatures in climatic chambers for 1, 15 and 30 days after preparation, respectively. An aliquot of the NEO was diluted in ultrapure water (1:9) to determine the size and distribution of the particles using a Zetasizer Nano ZS (Malvern Instruments); the refractive index was 1.60, and the samples were read in triplicate. Ten days after the NEO was obtained, it was characterized using dynamic light scattering (DLS), microelectrophoresis and atomic force microscopy (MAFM) to measure and determine the size distribution of the NEO, polydispersity index (PDI), and zeta potential (ζ).

An ultrahigh-resolution, field-free scanning electron microscope (SEM) (STEM-FEG) model CLARA was used to evaluate the morphology of NEOs (TESCAN). The NEO samples were prepared according to the methodology described by [45] with modifications. Initially, the samples were homogenized in a Vortex QL-901 shaker for 1 min. Then, an aliquot of 1 µL of each sample was spread evenly on a coverslip adhered to the stubs, which were stored in a container containing silica for drying and subjected to a gold bath to obtain the electron micrographs. The experimental design consisted of analyzing a single electron micrograph obtained at 100,000× magnification for each of the three storage temperatures. From particle size stability assessment, 30 nanospheres were randomly selected from each image and measured using ImageJ software Version 1.54p, resulting in a total of 90 measurements. The experiment was conducted in a completed randomized design with two factors: temperature (4, 25 and 40 °C) and storage period (1, 15 and 30 days).

### 4.5. Toxicity of Sprayed NEO of EO of E. uniflora on S. frugiperda via Potter’s Tower

The NEO was diluted in water to a concentration corresponding to the LC_50_ of the EO from *E. uniflora* (36.05 mg/mL). Next, 2 mL of this solution containing NEO was sprayed via a Potter tower on 72 h-old (2nd instar) *S. frugiperda* caterpillars, as described in subitem 4.3.1.

#### 4.5.1. Physiological Selectivity of NEO of *E. uniflora* for the Parasitoid *T. pretiosum*

The effects of NEO and NW on parasitoids during the immature stage were evaluated following the methodology described by [11], with modifications. The NEO was diluted in water to a concentration corresponding to the LC_50_ of the EO from *E. uniflora* (36.05 mg/mL). The NW, containing only the surfactants and water, was used as the negative control in these assays.

Forty female *T. pretiosum*, up to 24 h of age, were placed in glass tubes (8.5 cm high × 2.5 cm diameter) and fed droplets of pure honey, which was distributed on the walls of the tubes; the tubes were sealed with polyethylene plastic film (PVC) and perforated with an entomological pin for aeration. Approximately 125 fresh *E. kuehniella* eggs were attached to the ends of the blue card stock (5 cm long × 0.5 cm wide) using double-sided tape. These eggs were then unviability under ultraviolet light for 50 min [46] and offered to females of the maternal generation (F_0_) of the parasitoid for a period of 24 h. After this period, the females were discarded, and the cards containing the parasitized eggs (40 cards per treatment) were stored under the temperature and humidity conditions described in subitem 4.2, to obtain parasitoids of the F_1_ generation in the egg-larva and pupal stages (0–24 h and 168–192 h after parasitism) [47]. When the parasitoids reached these development stages inside the eggs of the alternative host, the eggs containing them were immersed in the NW and NEO solutions for 5 s [15]. The cartons with the treated eggs were stored under ambient conditions for approximately 1 h to eliminate excess moisture from their surfaces, then placed in new glass tubes and placed in an acclimatized chamber.

The experimental design was a factorial design (2 developmental periods × 2 treatments) with 40 replicates, each consisting of 1 tube with a carton of *E. kuehniella* eggs containing the parasitoid *T. pretiosum* in the egg–larva period or in the pupal stage. The effects of the treatments on the immature phase of parasitoids in the F_1_ generation were evaluated as a function of the number of emerged parasitoids and the number of males and females to calculate the sex ratio.

To evaluate the transgenerational effects of the treatments, 24 h after the birth of the F_1_ specimens, 20 females were randomly assigned to each treatment in glass tubes that contained honey droplets on the walls for feeding. Approximately 125 unfeasible and untreated *E. kuehniella* eggs were offered to these females; the eggs were arranged in blue cardboard cards (5 cm long × 0.5 cm wide) for a period of 24 h for parasitism. After this time, the cards with the parasitized eggs were placed in new tubes that were stored under controlled conditions. The assay was performed in accordance with a factorial design (2 development periods × 2 treatments). A total of 20 replicates per treatment were used, each consisting of 1 card and 1 female. Daily observations were performed to record the number of parasitized eggs and the survival of females in the F_1_ generation; however, to evaluate the transgenerational effects of the treatments on the insects in the F_2_ generation, the numbers of emerged parasitoids and females and males were quantified to determine the sex ratio of the offspring.

#### 4.5.2. Residual Effects of NEO on the Parasitism Capacity of *T. pretiosum*

Fresh *E. kuehniella* eggs were adhered to cartons and became unviable as described in subitem 4.5.1. Next, 40 females of *T. pretiosum* (generation F_0_) were individually placed in glass tubes and were fed droplets of pure honey, which was distributed on the walls of the tubes. The cards were treated by immersion in the NW and NEO solutions for 5 s [15], and at 12, 24 and 48 h after treatment, they were offered to the females for parasitism for 24 h. The cards that contained the eggs of the alternative parasitized host were placed in a climatic chamber, as described above, until the F_1_ insects emerged. The experiment followed a completely randomized factorial design (3 oviposition exposure times × 2 treatments), with 40 replicates per treatment. Each replicate consisted of one parasitoid female and one card containing treated host eggs. The number of parasitized eggs per female in the F_0_ generation and their longevity, as well as the numbers of emerged parasitoids and females and males, were evaluated to determine the sex ratio of the offspring (F_1_). The transgenerational effects of the treatments on the offspring of the F_1_ generation were quantified as a function of their parasitism, emergence and sex ratio (F_1_ generation).

### 4.6. Statistical Analysis

The normality and homoscedasticity of the data were verified using the Shapiro–Wilk and Bartlett tests. To confirm whether the biological replicates of the *S. frugiperda* trials could be combined, the homogeneity of variances between trials was assessed using Bartlett’s test. Since no significant heterogeneity was detected (*p* > 0.05), the data from the two replicates were pooled for subsequent analyses. The Kaplan–Meier non-parametric estimator was used to analyze insect survival over time. The median lethal time (LT_50_) was calculated and the curves compared using the Pairwise test (Survival packages). To determine the concentration-mortality response, the data was subjected to probit regression analysis using the drc package [48]. The log-likelihood ratio test was used to test the effect of NEO, followed by comparison (0.05). Quality was determined using a semi-normal plot with a simulation envelope. The NEO diameter data were analyzed using a tow-way ANOVA, followed by Tukey’s post hoc test (*p* < 0.05) to compare the mean particle sizes across different storage temperatures and periods. All analyses were performed using R^®^ software 4.5.2 version [49].

## 5. Conclusions

The EO and the NEO of *E. uniflora* demonstrated pronounced toxicity to *S. frugiperda*. The NEO exhibited physiological selectivity for the parasitoid *T. pretiosum* during its egg–larva period and pupa stage, with minimal effects on emergence and no adverse transgenerational impacts. However, residual exposure to treated host eggs negatively affected the parasitism capacity of adult females, indicating sublethal effects that may influence biological control performance depending on exposure timing. The NEO remained stable for up to 30 days at 25 °C, indicating satisfactory physiochemical robustness under controlled conditions. Overall, these results highlight the potential of this NEO for inclusion in IPM programs, provided that application strategies minimize adverse residual effects on beneficial insects. Further studies are required to assess safety for other non-target organisms, environmental persistence under field conditions, and the feasibility of large-scale production and commercialization.

## 6. Patents

The NEO was developed by the authors and is currently under patent protection (protocol No. NBR 10 2024 007294 4) [31].

## Figures and Tables

**Table 1:** Chemical composition of the essential oils of *Eugenia uniflora* essential oil.

N°	Compound	RRI ^a^	RRI ^b^	Area (%) ^c^ ± SD
				*E. uniflora* (Commercial)
1	β-Myrcene	991	988	1.00 ± 0.14
2	(Z)-β-Ocimene	1036	1032	1.64 ± 0.03
3	(E)-β-Ocimene	1045	1044	4.17 ± 0.54
4	σ-Elemene	1336	1335	1.15 ± 0.09
5	β–Elemene	1390	1389	4.95 ± 0.39
6	(E)-Caryophyllene	1416	1417	4.27 ± 0.38
7	γ-Elemene	1432	1434	2.53 ± 0.20
8	Germacrene D	1478	1480	5.03 ± 0.38
9	Curzerene	1497	1499	34.07 ± 1.93
10	δ-Cadinene	1522	1522	1.42 ± 0.09
11	Germacrene B	1554	1559	9.51 ± 0.53
12	Spathulenol	1575	1577	1.13 ± 0.07
13	Viridiflorol	1581	1592	0.73 ± 0.03
14	Selina-1,3,7(11)-trien-8-one	1631	1632	10.51 ± 0.11
15	Caryophylla-4(12),8(13)-dien-5α-ol	1634	1639	1.20 ± 0.07
16	Atractilone	1658	1657	1.33 ± 0.11
17	Germacrone	1694	1693	2.90 ± 0.12
18	Selina-1,3,7(11)-trien-8-one epoxide	1743	1747	2.56 ± 0.02
Monoterpenes hydrocarbons				6.81
Sesquiterpenes hydrocarbons				28.86
Oxygenated sesquiterpenes				54.43
Total (%)				90.10

RRI ^a^: Relative retention indices calculated against n-alkanes series (C8–C20) on the HP-5 MS column by elution order; RRI ^b^: Relative retention indices on an apolar column reported in the literature. Area (%) ^c^: average of the relative percentage area of the chromatographic peaks above 0.5%. SD, standard deviation (*n* = 3).

**Table 2:** Dose–response of essential oils of *Eugenia uniflora* sprayed via Potter’s tower on *Spodoptera frugiperda* caterpillars.

Treatment	N	*p*	LC_25_ (mg/mL)	LC_50_ (mg/mL)	LC_90_ (mg/mL)
EO *E. uniflora*	100	0.05	18.15 (9.29–34.62)	36.05 (25.87–52.44)	133.85 (85.85–208.39)

N: number of individuals.

**Table 3:** Mean physical diameter of individual nanoemulsion droplets of *Eugenia uniflora* essential oil measured by Scanning Electron Microscopy (SEM) following thermal stability and storage time tests.

Temperature	Storage Period		
	1 Day (nm)	15 Days (nm)	30 Days (nm)
4 °C	27.36 ± 1.04 aA	28.13 ± 1.03 aA	19.1 ± 0.47 bB
25 °C	26.06 ± 0.82 bA	22.70 ± 0.75 cB	31.13 ± 0.90 aA
40 °C	23.86 ± 0.76 bB	29.23 ± 0.98 aA	18.00 ± 0.60 cB

Means (± Standard Error) followed by the same lowercase letter in the row and uppercase in the column do not differ by the Tukey (*p* < 0.05).

**Table 4:** Emergence (%), parasitism (%) and sex ratio of *Trichograma pretiosum*, F_1_ generation, originating from *Ephestia kuehniella* eggs containing the parasitoid in the egg-larva period and in the pupal stage, contaminated with nanoemulsions of *Eugenia uniflora* essential oil or nanoemulsion white, and emergence and sex ratio of their descendants (F_2_ generation).

Treatment	Egg-Larva				Pupa			
	Emergence	Reduction	Sex Ratio	Parasitism	Emergence	Reduction	Sex Ratio	Parasitism
Generation F_1_								
NW	97.30 ± 2.53 aA	-	0.445 aA	27.00 ± 2.28 bB	86.20 ± 2.87 aA	2.60	0.442 aA	17.80 ± 3.56 aA
NEO	94.00 ± 1.52 aA	3.40	0.728 bB	21.70 ± 3.27 bB	88.50 ± 2.35 aA	-	0.645 bB	16.20 ± 3.26 aA
Generation F_2_								
NW	71.50 ± 2.50 bA	20.02	0.538 aA	-	66.20 ± 3.62 bA	12.90	0.593 aA	-
NEO	89.40 ± 2.91 bA	-	0.494 aA	-	76.00 ± 2.25 bA	-	0.517 aA	-

Means (± Standard Error) followed by the lowercase letter in the column (between treatments) and uppercase in the row (between development stages) do not differ statistically between them according to the Tukey test: Emergence F_1_ generation: (*p* = 0.6916, X^2^ = 333.31, GL = 98). Sex ratio F_1_ generation: (*p* = 0.6916, X^2^ = 333.31, GL = 98). Parasitism F_1_ generation (*p* = 0.0165, X^2^ = 1032.8, GL = 76). Emergence F_2_: (*p* = 0.084, X^2^ = 365.79, GL = 47). Sex ratio F_2_ generation (*p* = 0.082, X^2^ = 458; GL = 43). NW = nanoemulsion white—control, and NEO = nanoemulsion *E. uniflora* essential oil diluted to the LC_50_ = 36.05 mg/mL.

**Table 5:** Emergence (%), parasitism and sex ratio of parasitoids of the F_1_ generation, originating from F_0_ females of *Trichograma pretiosum* that parasitized *Ephestia kuehniella* eggs contaminated with nanoemulsions of *Eugenia uniflora* essential oil or nanoemulsion white, and offered 12, 24 and 48 h after treatment and emergence and sex ratio of their descendants (F_2_ generation).

Treatment	Contact 12 h After Treatment				Contact 24 h After Treatment				Contact 48 h After Treatment			
	Parasitism	Reduction	Emergence	Sex Ratio	Parasitism	Reduction	Emergence	Sex Ratio	Parasitism	Reduction	Emergence	Sex Ratio
Generation F_0_												
NW	19.50 ± 1.82 bB	-	-	-	11.80 ± 1.40 aB	-	-	-	11.70 ± 1.54 aB	-	-	-
NEO	3.16 ± 1.37 aA	83.80	-	-	1.02 ± 0.42 aA	91.40	-	-	2.18 ± 0.86 aA	81.40	-	-
Generation F_1_												
NW	21.00 ± 1.77 bA	-	85.00 ± 1.29 bB	0.399 aA	12.40 ± 2.75 aA	-	86.40 ± 1.02 bB	0.474 aA	10.10 ± 2.79 aA	-	83.10 ± 1.31 aB	0.940 bB
NEO	18.40 ± 2.12 aA	12.40	61.10 ± 3.26 aA	0.389 aA	8.80 ± 4.10 bA	29.03	68.10 ± 0.81 bA	0.333 aA	5.76 ± 2.49 bA	42.97	60.10 ± 1.67 aA	0.500 aA
Generation F_2_												
NW	-	-	77.00 ± 1.40 bA	0.934 aA	-	-	55.40 ± 2.61 bA	0.930 aA	-	-	38.40 ± 2.17 aB	0.930 aA
NEO	-	-	84.20 ± 1.40 bA	0.913 aA	-	-	43.10 ± 4.38 aA	1.00 aA	-	-	48.10 ± 3.05 aA	1.00 aA

Means (± Standard Error) followed by the lowercase letter in the column (between treatments) and uppercase in the row (between contact hours) do not differ statistically between them according to the Tukey test: Parasitism F_0_ generation: (*p* > 0.01; X^2^ = 2000; GL = 234). Emergence F_1_ generation (*p* = 0.447; X^2^ = 647.69; GL = 141). Sex ratio F_1_ generation (*p* > 0.01; X^2^ = 788.16; GL = 127). Parasitism F_1_ generation: (*p* > 0.01; X^2^ = 1268.1; GL = 104). Emergence F_2_: (*p* > 0.01; X^2^ = 631.53; GL = 82). Sex ratio F_2_ generation: (*p* = 0.05; X^2^ = 234.21; GL = 63). NW = nanoemulsion white—control, and NEO = nanoemulsion of *E. uniflora* essential oil diluted to the LC_50_ = 36.05 mg/mL.

## Data Availability

The original contributions presented in this study are included in the article. Further inquiries can be directed to the corresponding author.

## Abbreviations

The following abbreviations are used in this manuscript:

EO	Essential oil
EOs	Essential oils
NEO	Nanoemulsion of *Eugenia uniflora* essential oil
DLS	Dynamic Light Scattering
MAFM	Microelectrophoresis and Atomic Force Microscopy
PDI	Polydispersity Index
NW	Nanoemulsion White
SEM	Scanning Electron Microscope
IPM	Integrated Pest Management
